# Enhancing pediatric residents’ scholar role: the development of a Scholarly Activity Guidance and Evaluation program

**DOI:** 10.3402/meo.v20.27452

**Published:** 2015-06-08

**Authors:** Catherine M. Pound, Katherine A. Moreau, Natalie Ward, Kaylee Eady, Hilary Writer

**Affiliations:** 1 Children's Hospital of Eastern Ontario, Ottawa, Ontario, Canada; 2 Children's Hospital of Eastern Ontario Research Institute, Ottawa, Ontario, Canada

**Keywords:** research, postgraduate medical education, pediatrics

## Abstract

**Background:**

Research training is essential to the development of well-rounded physicians. Although many pediatric residency programs require residents to complete a research project, it is often challenging to integrate research training into educational programs.

**Objective:**

We aimed to develop an innovative research program for pediatric residents, called the Scholarly Activity Guidance and Evaluation (SAGE) program.

**Methods:**

We developed a competency-based program which establishes benchmarks for pediatric residents, while providing ongoing academic mentorship.

**Results:**

Feedback from residents and their research supervisors about the SAGE program has been positive. Preliminary evaluation data have shown that all final-year residents have met or exceeded program expectations.

**Conclusions:**

By providing residents with this supportive environment, we hope to influence their academic career paths, increase their research productivity, promote evidence-based practice, and ultimately, positively impact health outcomes.

Teaching medical residents the knowledge, skills, and attitudes they need to appraise and conduct research is essential to their development into well-rounded physicians. By equipping residents with the tools they need to succeed in research activities, residency programs can help promote evidence-based practice, advance the field of medicine, and ultimately, improve patient health outcomes. The General Standards of Accreditation of the Royal College of Physicians and Surgeons of Canada (RCPSC) require that residency programs ‘must be able to demonstrate that there are effective teaching programs in the critical appraisal of the medical literature’ and ‘be able to demonstrate that residents are able to conduct a scholarly project’ (1). In addition, the RCPSC pediatric-specific objectives of training state that all pediatric residents must significantly participate in a scholarly activity in order to be eligible to graduate from their residency program (2). The interpretation of what constitutes significant scholarly activity varies widely, with some programs accepting, for example, journal clubs, the teaching of other students, conference attendance, or resident research involvement as evidence of scholarly activity (3). Regardless of which form their scholarly activity takes, residents require guidance and evaluation to ensure they develop research skills, which carry benefits for clinical care, critical appraisal, clinical reasoning, and lifelong learning (4, 5).

Attempts to develop residents’ research skills have been described in both medical and surgical specialties (6–14). Yet, the successful conduct of research during residency training is not without its challenges. Residents have listed inadequate protected time, lack of previous research training, insufficient financial support, vague curricular requirements, and limited contact with ongoing research projects as perceived barriers (15). The lack of available mentors has also been identified as one of the key factors preventing residents from completing research during their clinical training (16, 17). Many pediatric programs encourage their residents to participate in or complete a research project during residency training. Although critical elements of successful programs have begun to emerge (18, 19), there is still an overall lack of evidence on how best to integrate a research training program into the culture of a residency training program (15, 18–20).

An informal review of Canadian pediatric residency programs demonstrated a widespread absence of structured programs to assist residents in the development of their research skills. While most Canadian pediatric residency programs have a coordinator to oversee their research activities, none, to our knowledge, have developed a formalized program with clear expectations and benchmarks. A needs assessment conducted in 2012 of both residents and faculty members who supervise resident research projects at our institution revealed that 32% of participating residents and 26% of participating supervisors believed that our center was not supportive of resident research in that it did not provide sufficient resources or training for research (21). Thus, there was a clear need for the development of a formal support system to foster resident research initiatives. In this article, we describe our Scholarly Activity Guidance and Evaluation (SAGE) program; a competency-based research program. This program provides ongoing academic mentorship and establishes benchmarks for pediatric residents, who are required to complete research projects as part of their residency training.

## Methods

In September 2012, we established the SAGE program for pediatric residents at a tertiary care pediatric academic health sciences center in Ontario, Canada. The program is based on the key competencies that residents must attain to fulfill the Scholar role, as described by the RCPSC. The Scholar role is one of seven roles that physicians are expected to develop over the course of their residency training (22). The key competencies that residents must demonstrate as part of the Scholar role include the ability to: 1) maintain and enhance professional activities through ongoing learning; 2) critically evaluate information and its sources, and apply this appropriately to practice decisions; 3) facilitate the learning of patients, families, students, residents, health professionals, the public, and others, as appropriate; and 4) contribute to the creation, dissemination, application, and translation of new medical knowledge and practices (23). The SAGE program focuses primarily on the development of residents’ abilities related to competencies 2 and 4 as described above.

## Program overview

The SAGE program includes two mandatory components. The first consists of in-person lectures and presentations on topics relevant to the development of scholarly projects (e.g., basic statistics, research design, research ethics, literature reviews, formulating research questions) and journal clubs. Since these activities have traditionally been part of the training of residents, they were seamlessly integrated into the SAGE program. The second and more innovative component of the program is designed to support and promote residents in the successful completion of their scholarly projects. The goal of this component of the program is to foster residents’ contributions to the creation, dissemination, application, and translation of new medical and educational knowledge and practices. By providing residents with the appropriate resources, guidance, and mentorship, along with regular assessments of their progress based on realistic and achievable benchmarks, we sought to improve residents’ scholarly abilities and develop accountability in terms of the completion of their projects.

Each resident is expected to participate in the program for the duration of their pediatric residency (typically 3–4 years). The first-year milestone consists of residents identifying a research idea, question(s), and project supervisor. Two worksheets need to be completed – one that helps them brainstorm a research idea and another that assists them in designing a question that accounts for the target population, intended intervention, comparison factor, and intended outcome. The second-year milestone consists of drafting a research proposal, which includes a literature review, research objective(s) and question(s), a description of the methodology, ethical considerations, dissemination plans, and a budget. The remaining milestones, which include carrying out the proposed research by meeting with content and methodology experts, submitting the projects to the required ethics board(s), applying for research funding (if needed), collecting and analyzing data, drafting a manuscript, and disseminating findings at academic conferences, are achieved over the subsequent 1 to 2 years of training (see Appendix A). The SAGE program provides structured support, guidance, mentorship to, and assessment of the residents throughout this process, as further detailed in the following sections.

## SAGE resources

A resident SAGE handbook is available in electronic and hardcopy format. The handbook describes the SAGE program and includes yearly milestones, timelines, worksheets, progress report forms, forms used by residents to request protected research time, links to grant and ethics application forms, as well as information on our newly developed online resident research training program (21). The online resident research training program includes four short online lessons on: 1) how to critically evaluate research literature; 2) how to write a research proposal; 3) how to submit an application for research funding; and 4) how to write a manuscript.

## SAGE rounds

SAGE rounds are open to all staff members at our institution and occur four times a year. This is a forum for residents to present their research-in-progress or nascent research ideas, and receive constructive feedback from program mentors and peers. Residents are instructed to keep their presentations informal, as a means of stimulating a fruitful and unintimidating discussion. Depending on the stage of the residents’ projects, these rounds can be used as an opportunity to bounce ideas off of the audience or discuss challenges.

## SAGE committee

This committee is chaired by the Scholarly Activity Coordinator and consists of 12 to 14 individuals. It includes representatives from the primary stakeholder groups in resident research – resident research supervisors, residents, community-based clinicians, residency program directors, and research staff with expertise in basic science, clinical research, and medical education research. Residents must submit a research progress report to this committee every six months that outlines the current state of their required scholarly projects and any challenges they are facing. The committee reviews the reports, provides written feedback to each resident, connects residents with content and methodological experts, and reviews their draft ethics and funding applications prior to submission.

## SAGE resident assessment

The SAGE committee uses an eight-item open-ended assessment form to provide formative feedback to each resident. This form is returned electronically to the resident and his/her research supervisor. The committee also assesses the residents’ progress to date, categorizing it as: ‘on track – no major concerns with progress’, ‘minor concerns with progress’, or ‘major concerns with progress’. If the committee has major concerns with a resident's progress, the Scholarly Activity Coordinator meets with the resident to discuss the perceived challenges and to jointly brainstorm solutions. In addition, the Scholarly Activity Coordinator meets individually with all first-year residents within the first six months of their residency training to provide guidance on the selection of a research topic and supervisor. Lastly, prior to writing their pediatric certification examinations, the Scholarly Activity Coordinator in consultation with the other members of the SAGE committee, determines if residents have met the established scholarly expectations. To graduate from the residency program, residents must have completed one of the following dissemination activities: 1) presentation of their project at the Institution's Annual Resident Research Day; 2) presentation of the project at a local, provincial, national, or international meeting; or 3) submission of a manuscript to a peer-reviewed journal.

## Results

Anecdotal feedback from residents and their research supervisors about the SAGE program has been positive. Preliminary evaluation data have also shown that, over the last two years, all final-year residents have met or exceeded the expectations of the SAGE program relating to the dissemination activities as stated above. In addition, the number of submissions to our Institutional Annual Resident Research Day has consistently increased, despite the fact that the number of residents enrolled in our local residency training program has not significantly risen. For example, in the 2011–12 academic year, we had seven resident research day submissions (out of 42 residents), compared to 12 submissions in the 2014–15 academic year (out of 43 residents).

Given these promising findings, we are now undertaking a formal and systematic evaluation of the SAGE program. The evaluation is structured around Guskey's five critical levels of evaluation (24) and will be used to strengthen the structure and content of the program. More specifically, we will evaluate the following: 1) level 1: residents’ and supervisors’ reactions to the program; 2) level 2: residents’ learning 3) level 3: organization support and change; 4) level 4: residents’ use of new knowledge and skills; and 5) level 5: residents’ learning outcomes. To guide this comprehensive evaluation, we have developed an evaluation framework, which includes the indicators, data sources, and data collection methods that we will use (see Appendix B).

## Conclusion

Our innovative SAGE program is designed to promote pediatric resident research success and foster CanMEDS Scholar competencies. As competency-based medical education has emerged as a priority topic in medical education (25), initiatives such as the SAGE program are of particular interest, as they specifically target scholar competencies, which have traditionally been found difficult to teach. The strength of the program lies in the easy access that residents have to a rich network of support, guidance, supervision, and mentorship, which are focused on the unique needs of each resident. It has been shown that residents exposed to research have an increased interest in academic careers (15). As such, we hope that, by providing residents with a supportive learning environment, we will influence their academic career paths, increase their research productivity, promote evidence-based practice, and ultimately, positively impact health outcomes.

## Appendix A

**Table:** Appendix A SAGE timeline and requirements

	Year 1	
Stage 1: Develop your idea	**You will …** **• Think about a research idea or question in the first 3 to 6 months of your residency** • Identify your scholarly project supervisor • Complete *Defining Your Question Worksheet* and submit it to SAGE (see worksheet on page 9) • Complete *PICO Worksheet* and submit it to SAGE (optional; see worksheet on page 10) • Perform a literature search or speak with CHEO expert (e.g., methodology expert from CRU)
	
	**SAGE will …** • Review your *Defining Your Question Worksheet* and optional *PICO Worksheet* • Identify challenges and suggest options for your project • Recommend CHEO content and methodology experts as well as relevant resources for your project **Note: Some scholarly questions may be deemed unacceptable (not feasible, unethical, prohibitively expensive, etc.) and the resident will return to Stage 1**

Year 2	

Stage 2: Refine your idea and prepare proposal	**You will …** • Prepare a draft 4 to 5 page proposal and submit it to SAGE for review. The proposal should include a literature review, clearly stated objectives, clear description of the methodology, description of the ethical consideration, dissemination plan, & budget
	**SAGE will …** • Review the full proposal, conduct a brief scientific review, and make one of three recommendations: (1) Approved; (2) Approved with minor revision; (3) Reconsider, revise and resubmit to SAGE

Years 2–4	

Stage 3: Revise and submit proposal to Research Ethics Boards (REBs) and CHEO RI	**You will …** • Revise your proposal (if necessary) • Meet with content and methodology experts to finalize your proposal (if necessary) • Submit your project (if necessary) to required REBs and to the CHEO RI if applying for resident research funding • Submit final proposal, copy of REB application and CHEO RI application (if necessary) to SAGE • Submit approval from REB and responses from CHEO RI to SAGE
Stage 4: Conduct project	**You will …** • Collect data, analyze data, write manuscript and disseminate findings (e.g., present at Resident Research Day)

## Appendix B

**Table:** Evaluation framework for SAGE program

Major evaluation questions	Indicators	Data sources	Data collection methods
Level 1: Residents’ and supervisors’ reactions			
Are the residents satisfied with the SAGE program?	- Residents’ opinions	- Residents	- Questionnaire
Are residents’ scholarly supervisors satisfied with the SAGE program?	- Scholarly supervisors’ opinions	- Scholarly supervisors	- Questionnaire
Level 2: Residents’ learning			
Do residents acquire the intended knowledge and skills to complete their research project?	- Residents’ opinions - # of completed projects - # of resident publications - # of presentations	- Residents - SAGE tracking logs	- Questionnaire - Document review
Level 3: Organization support & change			
Are sufficient resources available to the residents to support their research projects?	- Residents’ opinions - Scholarly supervisors’ opinions - Research Institute staff members’ opinions	- Residents - Scholarly supervisors - Research Institute staff members	- Interviews
Are residents’ research successes recognized and shared within our institution?	- Residents’ opinions - Scholarly supervisors’ opinions	- Residents - Scholarly supervisors	- Interviews
Level 4: Residents’ use of new knowledge and skills			
Are residents following the SAGE program's guidelines?	- # of submitted research question worksheets - # of submitted articles related to residents’ research ideas and methodology - # of submitted resident research proposals - # of submitted final resident research proposals	- SAGE tracking logs	- Document review
Are residents utilizing the research resources that have been developed or promoted by the SAGE program?	- # of residents using their dedicated research blocks - # of residents using the designated SAGE research training videos - # of residents attending SAGE rounds - # of residents accessing Research Institute services	- SAGE tracking logs - # of training video views - Research Institute staff members’ service logs	Document review
Level 5: Residents’ learning outcomes			
Is the SAGE program increasing residents’ research productivity?	- # of residents submitting grant applications - # of residents obtaining research grants - # of residents presenting at the annual resident research day - # of residents presenting at conferences - # of residents publishing in peer-reviewed journals	- SAGE tracking logs	- Document review
